# *Rasheedia* n. nom. (Nematoda, Physalopteridae) for *Bulbocephalus* Rasheed, 1966 (a homonym of *Bulbocephalus* Watson, 1916), with description of *Rasheedia heptacanthi* n. sp. and *R*. *novaecaledoniensis* n. sp. from perciform fishes off New Caledonia

**DOI:** 10.1051/parasite/2018033

**Published:** 2018-07-27

**Authors:** František Moravec, Jean-Lou Justine

**Affiliations:** 1 Institute of Parasitology, Biology Centre of the Czech Academy of Sciences Branišovská 31 370 05 České Budějovice Czech Republic; 2 Institut Systématique Évolution Biodiversité (ISYEB), Muséum National d’Histoire Naturelle, CNRS, Sorbonne Université, EPHE 57 rue Cuvier, CP 51 75005 Paris France

**Keywords:** Parasitic nematode, Taxonomy, Physalopteroidea, Marine fish, *Parupeneus*, *Dentex*, South Pacific

## Abstract

The nematode genus *Bulbocephalus* Rasheed, 1966 (Nematoda, Physalopteridae) was found to be a homonym of *Bulbocephalus* Watson, 1916 (Apicomplexa) and, therefore, a new name, *Rasheedia* n. nom., is proposed to substitute it. Based on light and scanning electron microscope studies of specimens collected from the digestive tract of perciform fishes off New Caledonia, two new species of *Rasheedia* are described: *R*. *heptacanthi* n. sp. from the Cinnabar goatfish *Parupeneus heptacanthus* (Mullidae) (type host) and *Dentex fourmanoiri* (Sparidae), and *R*. *novaecaledoniensis* n. sp. from the Indian goatfish *Parupeneus indicus* (Mullidae). These new species are mainly characterized by the number of anterior protrusible oesophageal lobes (two in *R*. *heptacanthi* and four in *R*. *novaecaledoniensis*), structure of the oesophagus and the lengths of spicules. An amended diagnosis of *Rasheedia* and a key to species of this genus are provided. Three previously described congeneric species are transferred to *Rasheedia* as *R*. *deblocki* (Le-Van-Hoa, Pham-Ngoc-Khue & Nguyen-Thi-Lien, 1972) n. comb., *R*. *inglisi* (Rasheed, 1966) n. comb. and *R*. *pseudupenei* (Vassiliadès & Diaw, 1978) n. comb. *Cestocephalus* Rasheed, 1966 [*genus inquirendum*], including *C*. *serratus* Rasheed, 1966 and *C*. *petterae* (Le-Van-Hoa, Pham-Ngoc-Khue & Nguyen-Thi-Lien, 1972) n. comb., should be considered to be separate from *Rasheedia*. The names *Pseudomazzia* Bilqees, Ghazi & Haseeb, 2005 and *P*. *macrolabiata* Bilqees, Ghazi & Haseeb, 2005, established for a nematode somewhat resembling *Rasheedia* spp., should be considered *nomina dubia*. *Rasheedia heptacanthi* n. sp. and *R*. *novaecaledoniensis* n. sp. are the first representatives of the Physalopteridae recorded from fishes in New Caledonian waters.

## Abstract (fr)

Le genre de nématode *Bulbocephalus* Rasheed, 1966 (Nematoda, Physalopteridae) a été trouvé homonyme de *Bulbocephalus* Watson, 1916 (Apicomplexa) et, par conséquent, un nouveau nom, *Rasheedia* n. nom., est proposé pour le remplacer. Sur la base d’études en microscopie optique et électronique à balayage de spécimens prélevés dans le tube digestif de poissons perciformes de Nouvelle-Calédonie, deux nouvelles espèces de *Rasheedia* sont décrites : *R. heptacanthi* n. sp. chez *Parupeneus heptacanthus* (Mullidae) (hôte-type) et *Dentex fourmanoiri* (Sparidae) et *R. novaecaledoniensis* n. sp. chez *Parupeneus indicus* (Mullidae). Ces nouvelles espèces sont principalement caractérisées par le nombre de lobes œsophagiens protrusibles antérieurs (deux chez *R. heptacanthi* et quatre chez *R. novaecaledoniensis)*, la structure de l’œsophage et la longueur des spicules. Un diagnostic modifié de *Rasheedia* et une clé pour les espèces de ce genre sont fournis. Trois espèces congénériques précédemment décrites sont transférées dans *Rasheedia* sous les noms *R. deblocki* (Le-Van-Hoa, Pham-Ngoc-Khué et Nguyen-Thi-Lien, 1972) n. comb., *R. inglisi* (Rasheed, 1966) n. comb. et *R. pseudupenei* (Vassiliadès & Diaw, 1978) n. comb. *Cestocephalus* Rasheed, 1966 [*genus inquirendum*], incluant *C. serratus* Rasheed, 1966 et *C. petterae* (Le-Van-Hoa, Pham-Ngoc-Khué et Nguyen-Thi-Lien, 1972) n. comb., doit être considéré comme distinct de *Rasheedia*. Les noms *Pseudomazzia* Bilqees, Ghazi & Haseeb, 2005 et *P. macrolabiata* Bilqees, Ghazi & Haseeb, 2005, établis pour un nématode ressemblant un peu à *Rasheedia* spp., devraient être considérés comme *nomina dubia*. *Rasheedia heptacanthi* n. sp. et *R. novaecaledoniensis* n. sp. sont les premiers Physalopteridae rapportés de poissons dans les eaux de Nouvelle-Calédonie.

## Introduction

Currently, there are two systems of classification for spirurine nematodes, one based principally on molecular data [7] and another based on morphological and biological data [3, 5, 6]. Nevertheless, since the former system seems to be premature at present [12], the suborder Spirurina of the order Spirurida of the latter system is followed in this paper. Of the ten superfamilies of the Spirurina, only four, the Gnathostomatoidea Railliet, 1895, Habronematoidea Chitwood & Wehr, 1932, Physalopteroidea Railliet, 1893 and Thelazioidea Skryabin, 1915, contain species that are parasitic as adults in freshwater, brackish-water or marine fishes, whereas members of the remaining six spirurine superfamilies are parasites exclusively of amphibians, reptiles, birds and mammals [5, 6]. Each of these superfamilies with fish parasites comprises a single family in which these fish nematodes are placed: Cystidicolidae Skryabin, 1946, Gnathostomatidae Railliet, 1895, Physalopteridae Railliet, 1893, and Rhabdochonidae Travassos, Artigas & Pereira, 1928 [12].

To date, the fauna of spirurine nematodes, as well as of other nematodes parasitizing marine fishes in New Caledonian waters, has not been well characterized. The spirurine family Gnathostomatidae is so far represented here by two species of *Echinocephalus* Molin, 1858, both parasites of elasmobranchs (rays) [17], whereas representatives of five genera belonging to the Cystidicolidae, i.e. *Ascarophis* van Beneden, 1871 (2 species), *Ascarophisnema* Moravec & Justine, 2010 (1 species), *Metabronema* Yorke & Maplestone, 1926 (1 species), *Metabronemoides* Moravec & Justine, 2010 (1 species), and *Spinitectus* Fourment, 1884 (1 species) have been reported from teleosts off New Caledonia and the nearby Chesterfield Islands [18–21]. The family Rhabdochonidae is represented here by a single species of *Johnstonmawsonia* Campana-Rouget, 1955 [15]. However, no species of the Physalopteridae has hitherto been recorded from New Caledonian fishes.

In 2009 and 2011, during extensive studies of the parasites of marine fishes in New Caledonian waters, physalopterid nematodes referable to the genus *Bulbocephalus* Rasheed, 1966 were collected from the digestive tract of the Cinnabar goatfish *Parupeneus heptacanthus* (Lacépède), the Indian goatfish *Parupeneus indicus* (Shaw) (both Mullidae, Perciformes), and the fish (no common name) *Dentex fourmanoiri* Akazaki & Séret (Sparidae, Perciformes). Closer examination of these nematodes revealed that they represent two new species. Results of the evaluation of these specimens are presented herein.

Whereas *P*. *indicus* and *P*. *heptacanthus* are tropical, reef-associated commercial fishes widespread in the Indo-Pacific region, *D*. *fourmanoiri* is a rare, deep-water fish with a limited distribution in the Southwest Pacific, occurring near the Chesterfield Islands and New Caledonia [9].

## Materials and methods

Fish were caught off New Caledonia by various means. The nematodes for morphological studies were fixed in hot 4% formalin or hot water then 70% ethanol. For light microscope examination (LM), they were cleared with glycerine. Drawings were made with the aid of a Zeiss microscope drawing attachment. Specimens used for scanning electron microscope examination (SEM) were postfixed in 1% osmium tetroxide (in phosphate buffer), dehydrated through a graded acetone series, critical-point-dried and sputter-coated with gold; they were examined using a JEOL JSM-7401F scanning electron microscope at an accelerating voltage of 4 kV (GB low mode). All measurements are in micrometres unless otherwise indicated. The fish nomenclature adopted follows FishBase [9].

## Results

Family Physalopteridae Railliet, 1893

Subfamily Proleptinae Schulz, 1927

### Genus *Rasheedia* n. nom.

urn:lsid:zoobank.org:act:70B0B83B-8347-4365-A99E-6841AEBA867C

Syn. *Bulbocephalus* Rasheed, 1966 (= a homonym to *Bulbocephalus* Watson, 1916)

Amended diagnosis: Body filiform. Cuticle slightly transversely striated. Anterior end of body with 2 lateral triangular pseudolabia, each provided with distinct terminal tooth, 2 cephalic papillae located dorso- and ventrolaterally, and lateral amphid. Oral aperture oval, dorsoventrally elongate, with smooth margin. Oesophagus divided into short anterior muscular and long posterior glandular regions. Muscular oesophagus consists of 3 portions: anterior, expanded portion modified to form protrusible pouch-like organ with 2–4 lobes; middle portion enclosed, along with nerve ring, by thin muscular sac-like structure; and narrow posterior portion. Excretory pore posterior to nerve ring. Deirids small, of irregular shape, with 2–5 prongs on distal end. Male with ventral, precloacal area rugosa formed by longitudinal ridges. Spicules unequal and dissimilar; right spicule shorter. Male posterior end with subventral caudal alae. Four pairs of preanal and 6 pairs of postanal papillae. Uterus of female amphidelphic. Vagina directed anteriorly from vulva. Fully developed eggs thick-walled, oval, containing larvae; eggs non-filamented. Type species: *R*. *inglisi* (Rasheed, 1966).

Etymology: The proposed new generic name is in honour of Suraiya Rasheed, a well-known Pakistani fish parasitologist who was the first to describe these interesting nematodes.

### *Rasheedia heptacanthi* n. sp. Figures 1–5

urn:lsid:zoobank.org:act:1521B92F-4A61-4BBD-82D3-8C37182141DA

**Figure 1.**
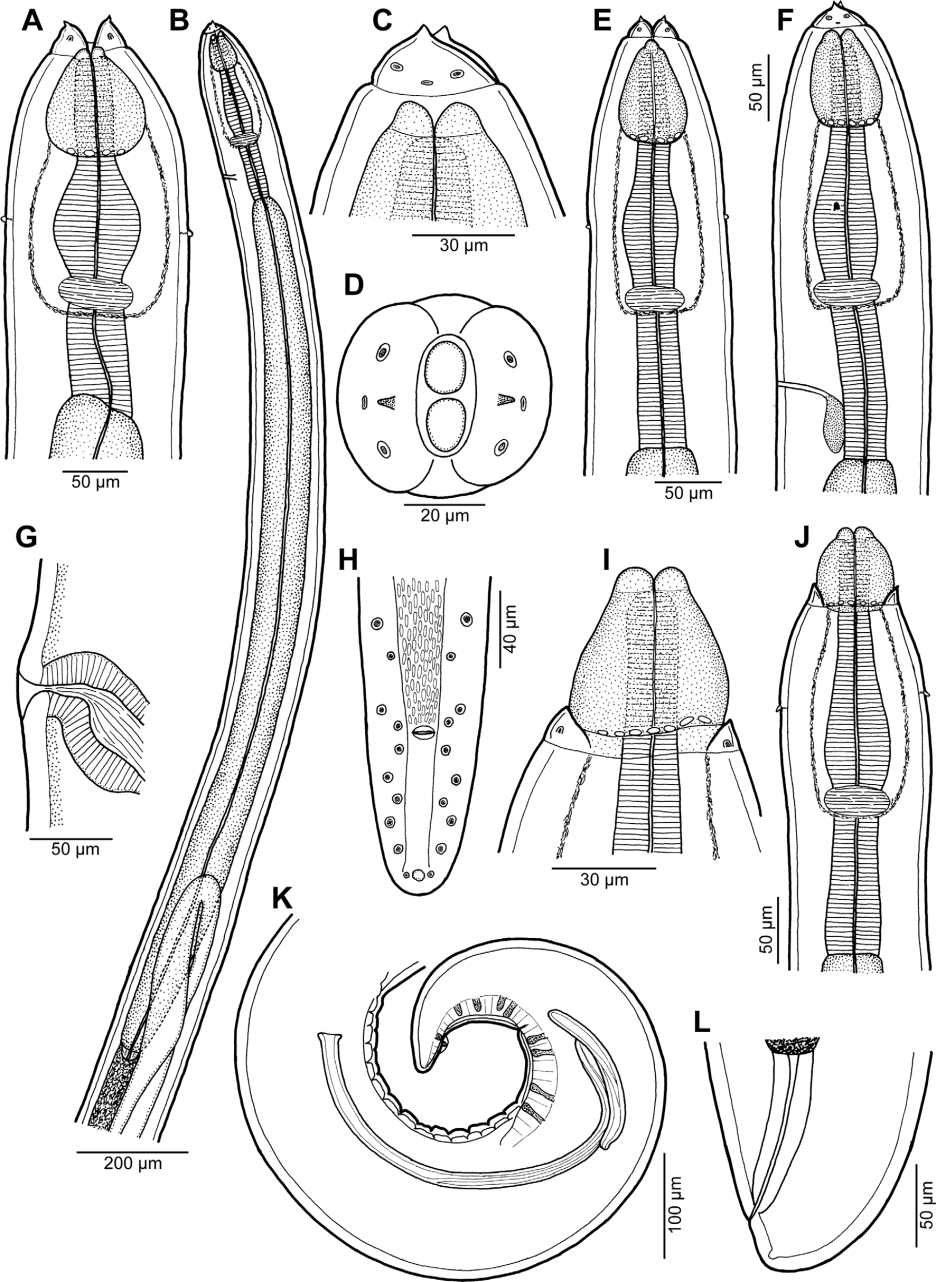
*Rasheedia heptacanthi* n. sp. (A) Anterior end of female, dorsoventral view; (B) anterior part of male body, lateral view; (C, D) cephalic end of male, lateral and apical views, respectively; (E, F) anterior end of male, dorsoventral and lateral views, respectively; (G) vulva, lateral view; (H) posterior end of male, ventral view; (I) cephalic end of male with extruded oesophageal lobes, dorsoventral view; (J) anterior end of male with extruded oesophageal lobes, dorsoventral view; (K, L) posterior end of male and female, respectively, lateral views (A–H, K, L from *P*. *heptacanthus*; I, J from *D*. *fourmanoiri*).

**Figure 2.**
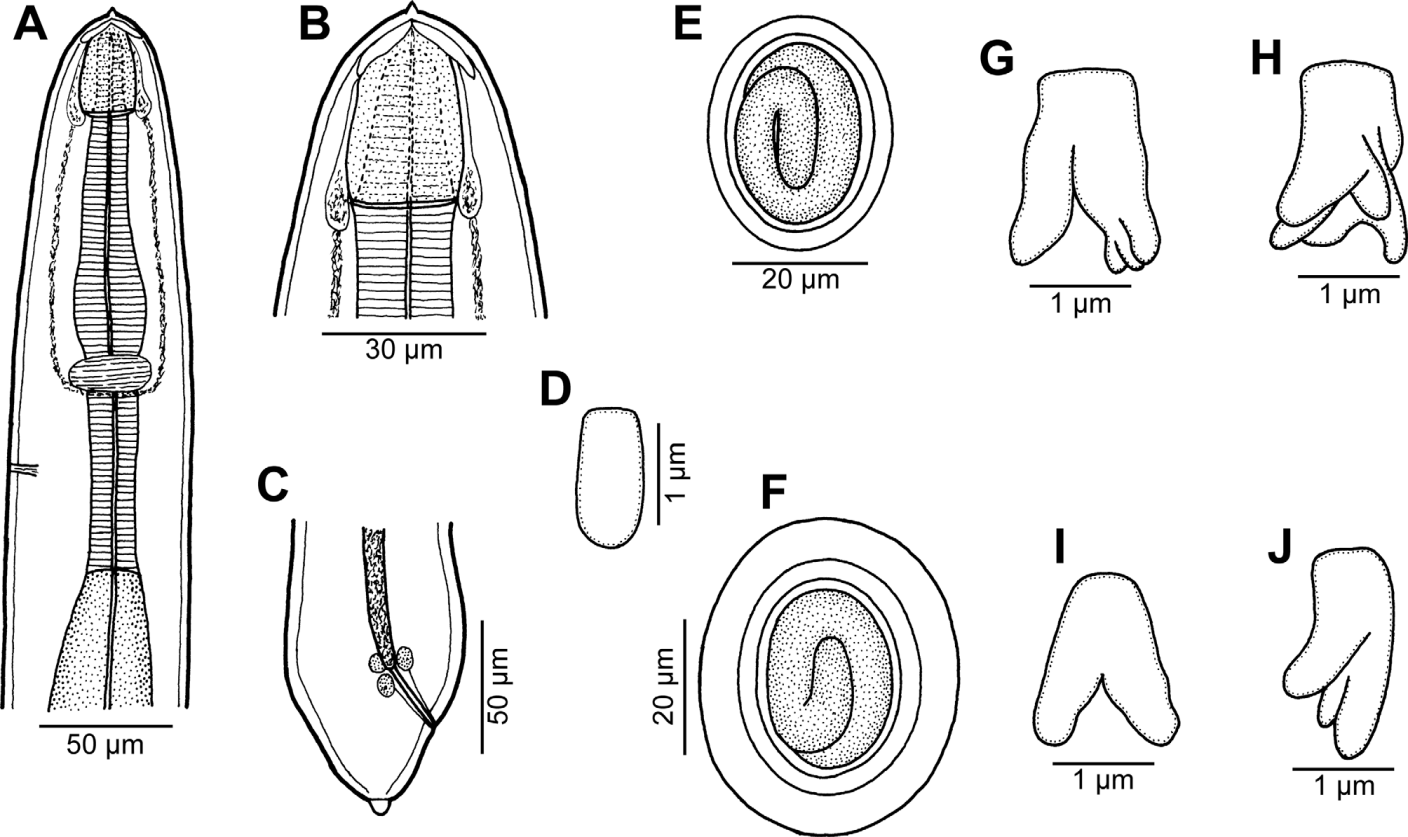
*Rasheedia heptacanthi* n. sp. (A–D) third-stage larva (A, anterior end; B, higher magnification of cephalic end; C, posterior end; D, deirid; all lateral views); (E) mature egg; (F) mature egg covered with additional thick layer; (G–J) morphological variations of deirids (A–D from *D*. *fourmanoiri*; E–J from *P*. *heptacanthus*).

**Figure 3.**
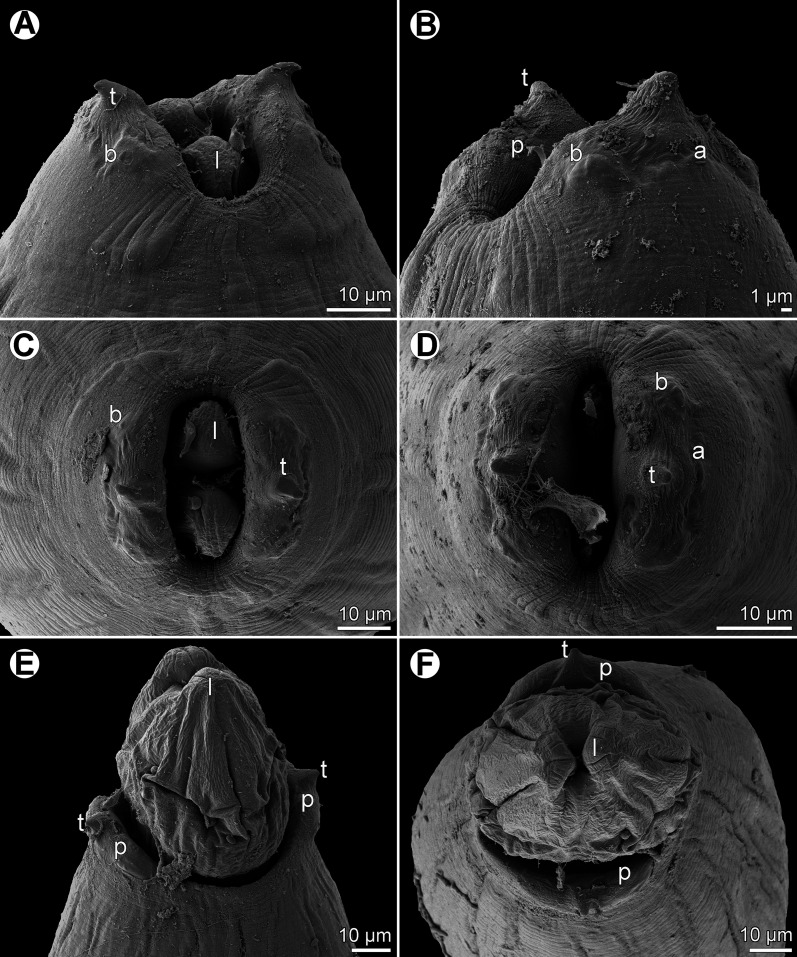
*Rasheedia heptacanthi* n. sp., scanning electron micrographs. (A, B) Cephalic end of female and male, subdorsoventral and sublateral views; (C, D) cephalic end of female and male, respectively, apical views; (E, F) cephalic end of male with extruded oesophageal lobes, dorsoventral and apical views. Abbreviations: (a) amphid; (b) cephalic papilla; (l) oesophageal lobe; (p) pseudolabium; (t) cephalic tooth. (A–D from *P*. *heptacanthus*; E, F from *D*. *fourmanoiri*).

**Figure 4.**
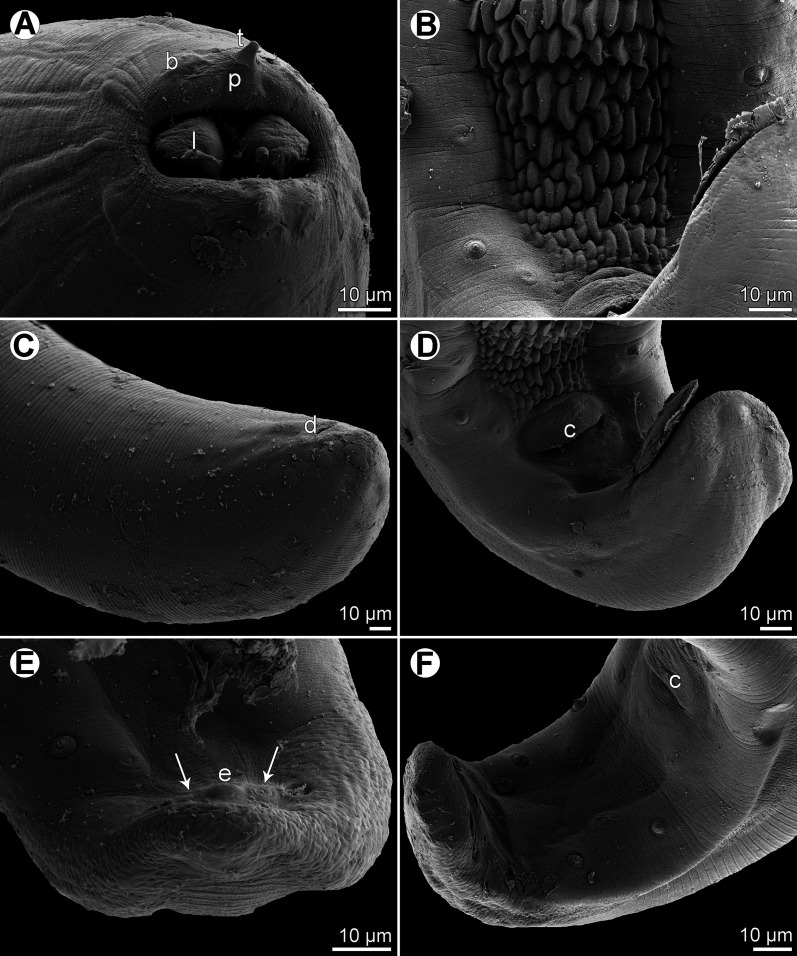
*Rasheedia heptacanthi* n. sp., scanning electron micrographs. (A) Cephalic end of male with weakly extruded oesophageal lobes, subapical view; (B) precloacal cuticular ridges, ventral view; (C) posterior end of female, sublateral view; (D) region of cloaca, ventral view; (E) posterior end of male tail, apical view (arrows indicate papillae of last postanal pair); (F) tail of male, ventrolateral view. Abbreviations: (b) cephalic papilla; (c) cloaca; (d) anus; (e) caudal protuberance; (l) oesophageal lobe; (p) pseudolabium; (t) cephalic tooth. (A, C, E, F from *P*. *heptacanthus*; B, D from *D*. *fourmanoiri*).

**Figure 5.**
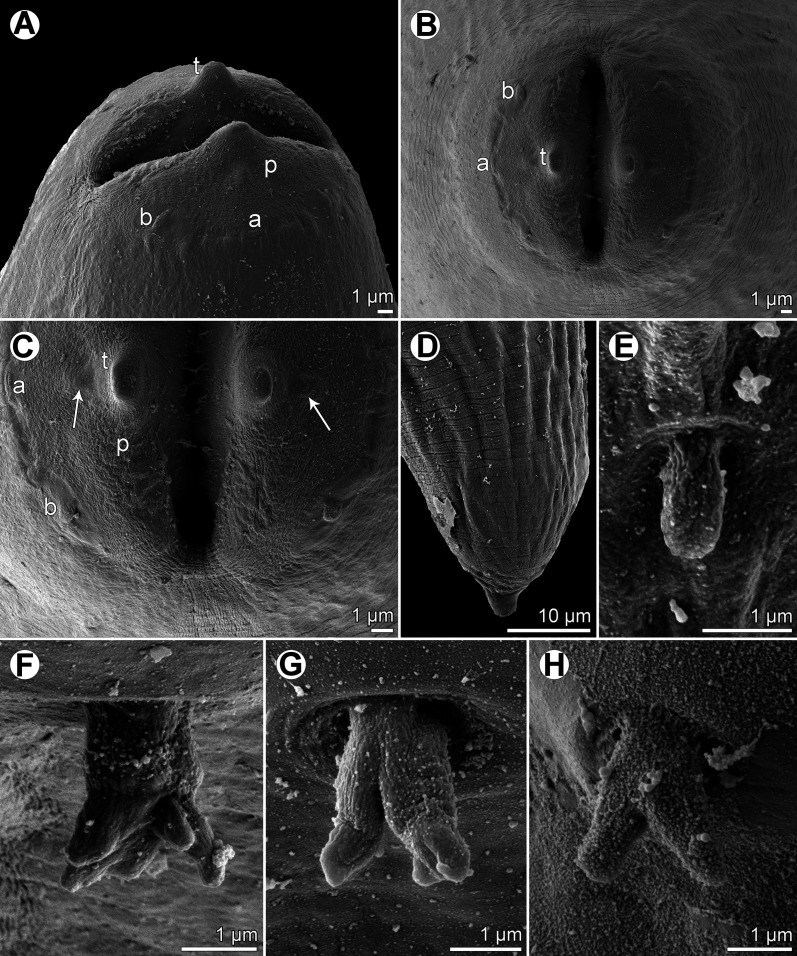
*Rasheedia heptacanthi* n. sp., scanning electron micrographs. (A, B) Cephalic end of third-stage larva, sublateral and apical views; (C) part of mouth (higher magnification) of third-stage larva, apical view (arrow indicates small elevation posterior to cephalic tooth); (D) caudal end of third-stage larva, lateral view; (E) deirid of third-stage larva; (F–H) morphological variability of deirids in adults. Abbreviations: (a) amphid; (b) cephalic papilla; (p) pseudolabium; (t) cephalic tooth. (A-E, H from *D*. *fourmanoiri*; F, G from *P*. *heptacanthus*).

Type host: Cinnabar goatfish *Parupeneus heptacanthus* (Lacépède) (Mullidae, Perciformes) (total body length 200 mm, weight 168 g). Parasitological record of fish: MNHN JC2984.

Other host: *Dentex fourmanoiri* Akazaki & Séret (Sparidae, Perciformes) (total body length 242 mm, weight 349 g). Parasitological record of fish: MNHN JC2992. A photograph of the fish is deposited in FishBase.

Site of infection: Digestive tract.

Type locality: (For *P. heptacanthus*) near Ilôt Pandanus, off Baie Maa, near Nouméa, New Caledonia (22°15′585S, 166°17′513E) (collected 18 June 2009).

Other locality: (For *Dentex fourmanoiri*) deep sea, external slope of the barrier reef, off Récif Toombo, near Nouméa, New Caledonia (22°34′841S, 166°27′612E, depth 200–350 m) (collected 2 July 2009).

Prevalence and intensity: *P*. *heptacanthus*: 1 fish infected/5 fish examined; 7 specimens. *D*. *fourmanoiri*: 1/1; 2.

Deposition of type specimens: Muséum National d’Histoire Naturelle, Paris, France (male holotype and 2 male paratypes, MNHN JNC 2984); Helminthological Collection, Institute of Parasitology, Biology Centre of the Czech Academy of Sciences, České Budějovice, Czech Republic (female allotype and 2 male paratypes mounted on SEM stub and 1 male paratype in vial, Cat. No. N–1161).

Etymology: The specific name of this nematode relates to the genitive form of the species name of the type host.

#### Description

*General:* Small, whitish nematodes with finely transversely striated cuticle (Figures 3A–3F, 4A–4F). Body somewhat narrowed towards anterior extremity (Figures 1A, 1B, 1E, 1F). Oral aperture dorsoventrally elongate, oval, rather large, surrounded by 2 triangular lateral pseudolabia, each with distinct terminal tooth; each pseudolabium bears 2 submedian (dorsolateral and ventrolateral) cephalic papillae and small lateral amphid (Figures 1A, 1C, 1D, 3A–3D, 4A). Oesophagus divided into short anterior muscular and long posterior glandular regions (Figure 1B). Muscular oesophagus consists of 3 portions: anterior, expanded portion modified to form protrusible pouch-like organ with 2, dorsal and ventral, lobes and with granular structures at bottom; middle, distinctly widened portion enclosed, along with nerve ring, by thin muscular sac-like structure; and narrow posterior portion (Figures 1A–1F, 1I, 1J, 3A, 3C, 3E, 3F). Deirids small, with 2–5 distal prongs, situated approximately at mid-way between posterior end of anterior protrusible pouch-like organ and nerve-ring (Figures 1A, 1E, 1F, 1J, 2G–2J, 5F–5H). Excretory pore approximately at level of mid-way between nerve ring and anterior end of glandular oesophagus (Figures 1B and 1F). Tail of both sexes with rounded tip.

*Male* (6 specimens from *P*. *heptacanthus*, with measurements of holotype in parentheses; measurements of 1 specimen from *D*. *fourmanoiri* in brackets): Length of body 4.79–5.32 (4.94) [4.60] mm, maximum width 163–177 (177) [150]. Pseudolabia 18–21 (18) [not measured] long. Entire oesophagus 2.10–2.36 (2.19) [2.7] mm long, representing 41–44% (44%) [53%] of body length; anterior, protrusible part of muscular oesophagus 75–90 (84) [75] long and 60–63 (63) [69] wide; middle portion of muscular oesophagus 150–180 (150) [168] long, maximum width 45–57 (57) [48]; posterior portion of muscular oesophagus 114–123 (120) [108] long, width 39–42 (39) [39]; glandular oesophagus 1.74–1.97 (1.84) [2.11] mm long and 93–105 (93) [96] wide; muscular sac-like structure enclosing middle portion of muscular oesophagus and nerve-ring 150–180 (150) [168] long and 66–87 (84) [69] wide. Deirids, nerve ring and excretory pore 165–186 (180) [165], 240–264 (240) [240] and 306–369 (324) [324] from anterior extremity, respectively. Caudal end spirally coiled, provided with lateral alae supported by 4 pairs of subventral pedunculate preanal papillae arranged in couples, and 5 single pairs of rather large, pedunculated subventral postanal papillae; an additional pair of small postanal sessile papillae situated ventrally slightly posterior to level of last subventral postanal pair (Figures 1H, 1K, 4E, 4F). Phasmids not found. Ventral surface between ventral postanal papillae elevated to form small median protuberance (Figures 1H, 4E). Ventral precloacal surface with 12 longitudinal tessellated ridges (area rugosa) (Figures 1H, 1K, 4B, 4D). Spicules unequal and dissimilar (Figure 1K); left spicule 435–489 (465) [474] long; its shaft 165–189 (189) [171] long, representing 38–42% (41%) [36%] of spicule length; right spicule boat-shaped, 153–156 (156) [153] long; length ratio of spicules 1:2.8–3.2 (1:3.0) [1:3.1]. Length of tail 159–192 (180) [153].

*Female* (1 ovigerous specimen, allotype, from *P*. *heptacanthus*): Length of body 6.58 mm, maximum width 258. Pseudolabia 18 long. Entire oesophagus 2.42 mm long, representing 37% of body length; anterior, protrusible part of muscular oesophagus 99 long and 87 wide; middle portion of muscular oesophagus 135 long, maximum width 72; posterior portion 81 long, width 60; glandular oesophagus 2.11 mm long and 144 wide; muscular sac-like structure enclosing middle part of muscular oesophagus and nerve ring 135 long and 120 wide. Deirids, nerve ring and excretory pore 180, 231 and 285 from anterior extremity, respectively. Vulva situated 4.39 mm from anterior end of body, at 67% of body length; vulval lips slightly elevated (Figure 1G). Vagina muscular, directed anteriorly from vulva and short ovijector. Uteri containing numerous oval, thick-shelled, embryonated (larvated) eggs; eggs 36–39 × 27–30, with wall 3 thick. Eggs located in uterus near vulva covered by additional, light-coloured thick layer, appearing as kind of capsule with enclosed proper egg; size of eggs including such capsule 48–54 × 36–39. Tail very short, 21, with rounded tip (Figures. 1L, 4C).

*Third-stage larva* (1 specimen from *D*. *fourmanoiri*): Length of body 2.23 mm, maximum width 82. Pseudolabia 9 long; small cuticular elevation present posterior to cephalic tooth (Figures 2A, 2B, 5A–5C). Entire oesophagus 1.25 mm long, representing 56% of body length; anterior, protrusible part of muscular oesophagus 39 long and 27 wide (Figures 2A, 2B); middle portion of muscular oesophagus 120 long, maximum width 3; posterior portion 75 long, width 21; glandular oesophagus 1.02 mm long and 51 wide; muscular sac-like structure enclosing middle part of muscular oesophagus and nerve ring 120 long and 45 wide (Figure 2A). Deirids simple, with rounded distal end (Figures 2D, 4E). Deirids, nerve ring and excretory pore 111, 150 and 189 from anterior extremity, respectively. Tail 57 long, rounded, with small cuticular knob at tip (Figures 2C, 5D).

#### Remarks

According to the key to spiruride nematodes of Chabaud [5, 6], the present nematodes belong to the genus *Bulbocephalus* Rasheed, 1966. However, as we found, this name is pre-occupied by *Bulbocephalus* Watson, 1916, a genus of gregarines (Apicomplexa, Eugregarinorida, Stylocephalidae) [26], meaning that the former generic name becomes a homonym for the latter. Therefore, we propose a new generic name, *Rasheedia* n. nom., for nematodes previously listed in *Bulbocephalus*. These are transferred to *Rasheedia* as *R*. *deblocki* (Le-Van-Hoa, Pham-Ngoc-Khue & Nguyen-Thi-Lien, 1972) n. comb., *R*. *inglisi* (Rasheed, 1966) n. comb., and *R*. *pseudupenei* (Vassiliadès & Diaw, 1978) n. comb.

From marine fishes of Pakistan, Rasheed [24] inadequately described two species of interesting spiruroid nematodes with protrusible anterior oesophageal lobes, for which she established the new monotypic genera *Bulbocephalus* (type species *B*. *inglisi*) and *Cestocephalus* Rasheed, 1966 (type species *C*. *serratus* Rasheed, 1966). Subsequently, based on similar nematodes collected from fishes in South Vietnam, Le-Van-Hoa, Pham-Ngoc-Khue & Nguyen-Thi-Lien [11] synonymized *Cestocephalus* with *Bulbocephalus*, which was followed by Chabaud [5, 6] and subsequent authors; they also designated *C*. *serratus* of Rasheed [24], inadequately described from a single female, to be a *species inquirenda*. However, in accordance with the international rules, the genus is objectively determined by its type species and, consequently, *Cestocephalus* becomes a *genus inquirendum* and cannot be considered a synonym of *Bulbocephalus* (= *Rasheedia*). Therefore, the Vietnamese species similar to *C*. *serratus* should be tentatively reported as *Cestocephalus petterae* (Le-Van-Hoa, Pham-Ngoc-Khue & Nguyen-Thi-Lien, 1972) n. comb. Since the cephalic structures of both *C*. *serratus* and *C*. *petterae* are insufficiently studied, it is reasonable to consider *Rasheedia* and *Cestocephalus* [*genus inquirendum*] as possibly different genera. In contrast to *Rasheedia* spp., both representatives of *Cestocephalus* are allegedly characterized by the presence of three large oesophageal lobes resembling bothridia of cestodes, which form a hood or umbrella-like structure on the cephalic end, and by a deeply transversely striated cuticle appearing from the side to have sharp edges or serrations.

It follows from the above reasoning that, to date, there are three species of *Rasheedia*: 1) *R*. *inglisi* described from two males collected from an unidentified fish species (known in Sindhi as “Jhikar”) and two females from *Eleutheronema tetradactylum* (Shaw) (Polynemidae) off Pakistan [24]; from Pakistan, a single female of *R*. *inglisi* was also reported from *Psettodes erumei* (Bloch & Schneider) (Psettotidae, Pleuronectiformes) [1, 2], but this record is probably based on a misidentification; 2) *R*. *deblocki* from *E*. *tetradactylum* off South Vietnam [11]; and 3) *R*. *pseudupenei* from *Pseudupeneus prayensis* (Cuvier) (Mullidae) off Senegal [25].

The original description of *R*. *inglisi* is inadequate, especially regarding the cephalic structures, and, as mentioned by Le-Van-Hoa et al. [11], the presence of pseudolabia was evidently overlooked by Rasheed [24]. Moreover, considering that the specimens were collected from two different species of hosts, it is not certain that the available nematode males and females belonged to one and the same species. According to Le-Van-Hoa et al. [11], *R*. *deblocki* is very similar to *R*. *inglisi*, differing from it only in the presence of a single point (instead of allegedly three small points) on the female tail tip, ventral precloacal cuticular ridges in the male and in somewhat greater measurements. However, it is highly probable that Rasheed [24] considered a pair of phasmids, situated near the female tail tip as illustrated for *R*. *deblocki*, to be terminal points and she apparently overlooked the presence of precloacal ridges in *R*. *inglisi*. Since both of these nematode species were collected from *E*. *tetradactylum* in the Indo-West Pacific region, it is highly probable that subsequent studies will prove their conspecificity; some biometrical differences may well be within the intraspecific variability of a single species. However, for the time being and until new material is available, we consider *R*. *inglisi* and *R*. *deblocki* to be separate species.

*Rasheedia heptacanthi* n. sp. is easily distinguished from *R*. *inglisi* and *R*. *deblocki* by distinctly smaller body measurements, the longer left spicule and the unusual structure of eggs. It differs from *R*. *pseudupenei* mainly in having longer spicules, different structure of eggs and in the geographical distribution (see the key to species of *Rasheedia* at the end of Discussion). Although *R*. *inglisi*, *R*. *deblocki* and *R*. *pseudupenei* are reported to possess three anteriorly protruding oesophageal lobes, there are only two, one dorsal and one ventral, lobes in the new species, as revealed by SEM examination.

### *Rasheedia novaecaledoniensis* n. sp. Figures 6 and 7

urn:lsid:zoobank.org:act:98BC4949-A069-43CB-9C74-48A265DAE3CD

**Figure 6.**
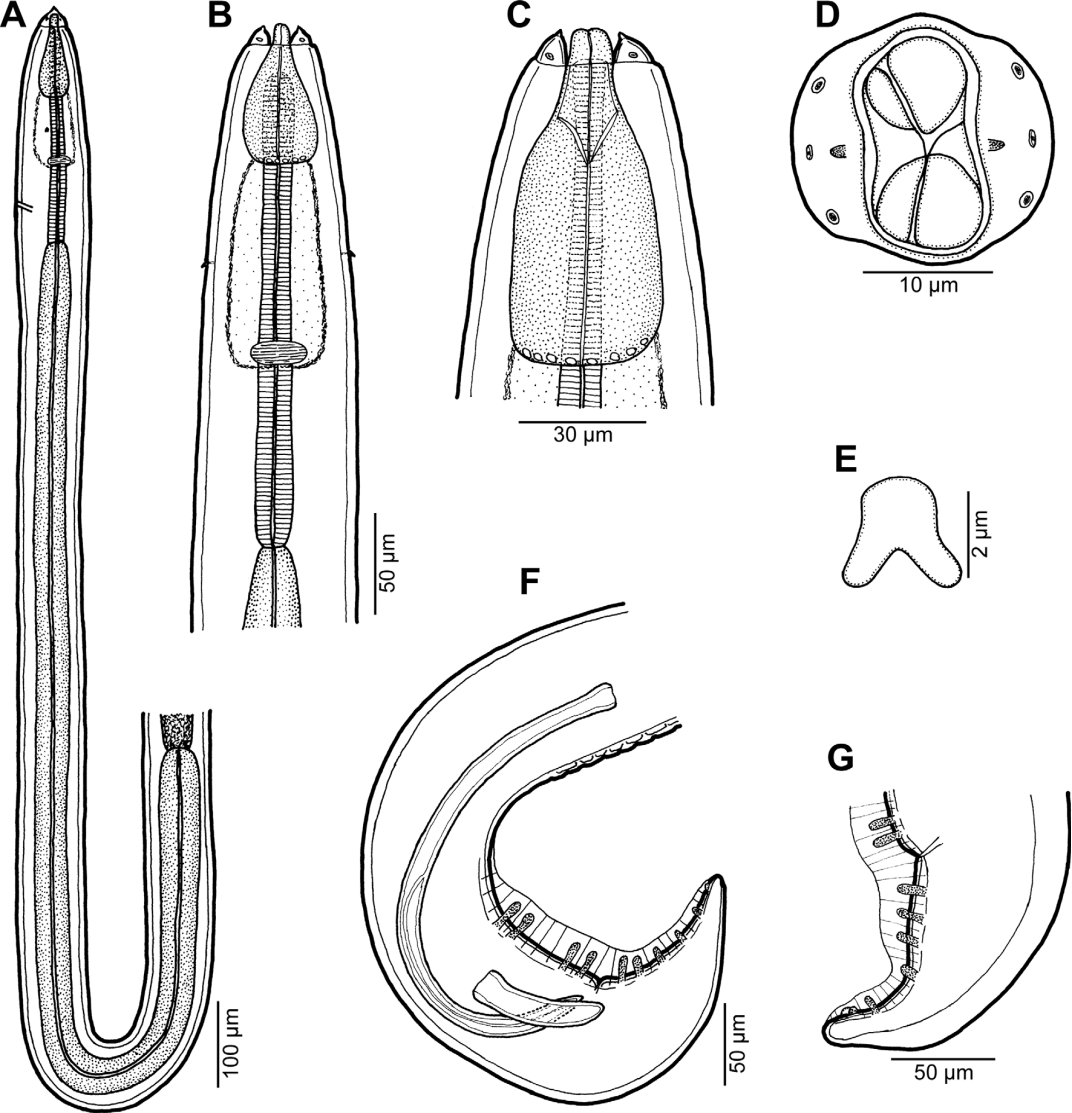
*Rasheedia novaecaledoniensis* n. sp. from *P*. *indicus*, male. (A) Anterior part of body, lateral view; (B) anterior end, dorsoventral view; (C, D) cephalic end, dorsoventral and apical views, respectively; (E) deirid; (F) posterior end, lateral view; (G) tail, lateral view.

**Figure 7.**
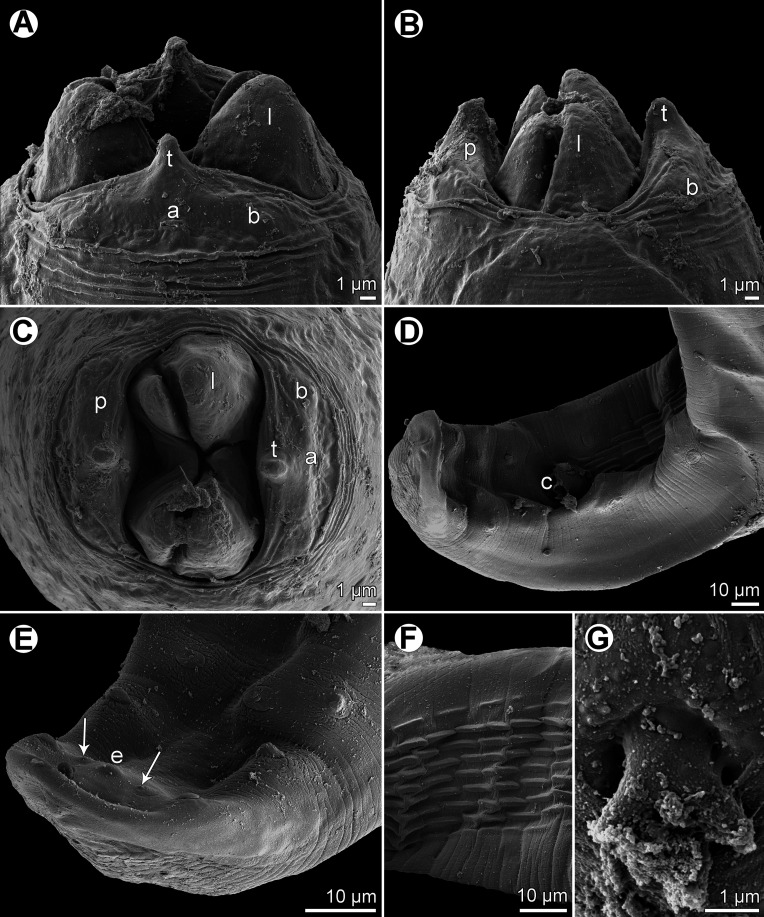
*Rasheedia novaecaledoniensis* n. sp. from *P*. *indicus*, scanning electron micrographs of male. (A–C) Cephalic end, lateral, dorsoventral and apical views, respectively; (D) tail, ventrolateral view; (E) posterior end of tail, ventrolateral view (arrows indicate papillae of last postanal pair; (F) precloacal cuticular ridges, ventral view; (G) deirid. Abbreviations: (a) amphid; (b) cephalic papilla; (c) cloaca; (e) caudal protuberance; (l) oesophageal lobe; (p) pseudolabium; (t) cephalic tooth.

Type host: Indian goatfish *Parupeneus indicus* (Shaw) (Mullidae, Perciformes) (total body length 270 mm, weight 425 g). Parasitological record of fish: MNHN JC3305.

Site of infection: Digestive tract.

Type locality: Fishmarket of Nouméa, New Caledonia (collected 4 February 2011).

Prevalence and intensity: 1 fish infected/5 fish examined; 2 specimens.

Deposition of type specimen: Helminthological Collection, Institute of Parasitology, Biology Centre of the Czech Academy of Sciences, České Budějovice, Czech Republic (male holotype mounted on SEM stub, Cat. No. N–1162). Larval specimen not deposited, intended for sequencing.

Etymology: The scientific name *novaecaledoniensis* relates to the country, i.e. New Caledonia, near the coast of which the fish host of this nematode was collected.

#### Description

*Male* (1 specimen): Small, whitish nematode with finely transversely striated cuticle (Figures 7A–7F). Body somewhat narrowed towards anterior extremity (Figures 6A–6C). Length of body 4.05 mm, maximum width 99. Oral aperture dorsoventrally elongate, oval, rather large, surrounded by 2 triangular lateral pseudolabia, each with distinct terminal tooth; pseudolabia 21 long; each pseudolabium bears 2 submedian (dorsolateral and ventrolateral) cephalic papillae and small lateral amphid (Figures 6A–6D, 7A–7C). Oesophagus divided into short anterior muscular and long posterior glandular regions (Figure 6A). Muscular oesophagus consists of 3 portions: anterior, expanded portion modified to form protrusible pouch-like organ with 4 asymmetrical submedian lobes and with granular structures at bottom; middle narrow portion enclosed, along with nerve ring, by thin muscular sac-like structure; and narrow posterior portion (Figures 6A–6C, 7A–7C). Entire oesophagus 1.73 mm long, representing 43% of body length; anterior, protrusible portion of muscular oesophagus 66 long and 18 wide; middle portion of muscular oesophagus 126 long, 12 wide, posterior portion 99 long, width 18; glandular oesophagus 1.44 mm long and 39 wide. Muscular sac-like structure enclosing middle portion of muscular oesophagus and nerve ring 126 long and 48 wide. Deirids small, bifurcate, situated approximately at mid-way between posterior end of anterior protrusible pouch-like organ and nerve ring (Figures 6B, 6E, 7G). Excretory pore approximately at level of mid-way between nerve ring and anterior end of glandular oesophagus (Figure 6A). Deirids, nerve ring and excretory pore 117, 171 and 192 from anterior extremity, respectively. Caudal end spirally coiled, provided with lateral alae supported by 4 pairs of subventral pedunculate preanal papillae arranged in couples, and 5 single pairs of subventral postanal papillae, which are rather large, pedunculate; an additional pair of small postanal sessile papillae situated ventrally slightly posterior to level of last subventral postanal pair (Figures 6F, 6G, 7E). Ventral surface between ventral postanal papillae elevated to form small caudal protuberance (Figure 7E). Ventral precloacal surface with about 9 longitudinal tessellated ridges (area rugosa) (Figures 6F, 7D, 7F). Spicules unequal and dissimilar (Figure 6F); left spicule 381 long; its shaft 186 long, representing 49% of spicule length; right spicule boat-shaped, 87 long; length ratio of spicules 1:4.38. Length of tail 102.

*Male fourth-stage larva* (1 specimen): Length of body 2.87 mm, maximum width 87. Pseudolabia 12 long. Entire oesophagus 1.26 mm long, representing 44% of body length; anterior, protrusible part of muscular oesophagus 36 long and 18 wide; middle portion of muscular oesophagus 126 long and 33 wide; posterior portion 99 long, width 18; glandular oesophagus 1.01 mm long and 45 wide. Muscular sac-like structure enclosing middle part of muscular oesophagus and nerve ring 126 long and 33 wide. Nerve ring and excretory pore 144 and 183 from anterior extremity, respectively; deirids not located. Developing spicules weakly sclerotized; left spicule 195 long, length of right spicule 87. Caudal papillae not yet developed. Tail 72 long, rounded. Larva starting last moult, still inside sheathed old cuticle.

*Female*: Not known.

#### Remarks

This new species differs from other congeners in possessing four (instead of two or three) anterior protrusible oesophageal lobes. In addition, *R*. *inglisi* and *R*. *deblocki* have much larger body measurements and they are parasites of the Polynemidae (*vs.* Mullidae). In contrast to *R*. *heptacanthi* n. sp., the muscular oesophagus in the portion anterior to the nerve ring of *R*. *novaecaledoniensis* n. sp. is not expanded and the spicules are distinctly shorter.

Deirids of *R*. *pseudupenei* are located much more anteriorly to the level of the nerve ring as compared with those in *R*. *novaecaledoniensis* n. sp. and both species differ in the genus of hosts and in distant geographical regions (North Atlantic *vs.* South Pacific) (see the key to species of *Rasheedia* at the end of Discussion).

From the intestine of the same host species (*P*. *indicus*) in the Indian Ocean off Somalia, another spirurine nematode, *Ascarophis parupenei* Moravec, Orecchia & Paggi, 1988 (Cystidicolidae, Habronematoidea) was described [23]. However, the cephalic structure of this nematode is very different from that of *R*. *novaecaledoniensis* n. sp. and the spicules are much longer (533–600 μm and 150–171 μm *vs.* 381 μm and 87 μm).

## Discussion

The present study shows that the morphology of *Rasheedia* spp. is very unusual among parasitic nematodes, namely in possessing the anterior portion of the muscular oesophagus modified to form a protrusible pouch-like organ with anterior lobes and the middle portion of the muscular oesophagus enclosed in a thin-walled muscular sac-like structure. These features are already present in the third-stage larva of *R*. *heptacanthi* examined (Figures 2A, 2B). All previously described species of *Rasheedia* (reported as *Bulbocephalus*) were studied only by LM and probably due to the very limited numbers of available specimens, and the fact that some morphological features are not easily visible using LM, the existing species descriptions are evidently incomplete and may even be somewhat misleading.

The present study of two species of *Rasheedia* by SEM, used for the first time for nematodes of this genus, made it possible to describe in detail the cephalic structures. Whereas the presence of three protrusible oesophageal lobes was reported for all the three previously described species of *Rasheedia*, the SEM micrographs revealed two or four lobes in the two newly established species. Although Rasheed [24] did not mention the presence of deirids in *R*. *inglisi*, these were reported (but not illustrated) for *R*. *deblocki* [11] and for *R*. *pseudupenei* [25], but their shape was not described. The SEM micrographs of *R*. *heptacanthi* n. sp. showed considerable intraspecific variability in the shape of deirids in adults of this species, which may be from bifurcate (Figures 2I, 5H) to possessing up to five distal prongs (Figures 2G, 2H, 2J, 5F, 5G). The shape of deirids is usually stable within the same species in spirurids and is frequently considered to be a reliable interspecific taxonomic feature in some groups, for example in species of *Rhabdochona* Railliet, 1916 (Rhabdochonidae, Thelazioidea) [13]. However, in contrast to adults of *R*. *heptacanthi* n. sp., the conspecific third-stage larva was found to possess simple, rounded deirids (Figures 2D, 5E). The presence of a small ventral median caudal protuberance, found in the males of both new species of *Rasheedia*, was not previously reported for representatives of this genus. Such a caudal protuberance has so far been observed only in some cystidicolid nematodes [8, 15, 16, 19, 22].

A remarkable feature of *R*. *heptacanthi* n. sp. is that the fully developed eggs located in the uterus near the vulva have an additional thick outer layer, appearing like a capsule enclosing the true egg (Figure 2F). As far as the authors know, among fish nematodes, mature eggs covered by an additional thick outer layer have only been described in a few species of capillariids (Capillariidae) parasitizing marine fishes, for example *Gessyella latridopsis* (Johnston & Mawson, 1945) or *Capillaria appendigera* Moravec & Barton, 2018 from Australian waters [10, 14], but these are not known in species of the Spirurida.

The third-stage larva of *R*. *heptacanthi* n. sp. is the first infective larva of *Rasheedia* described so far. Its morphology is very similar to that of conspecific adults, but its deirids are simple and the tail tip is provided with a cuticular knob, resembling thus third-stage larvae of many other spiruride nematodes parasitizing fishes as adults. It can be assumed that, as in most spirurides parasitizing marine fishes, the life cycles of *Rasheedia* spp. involve arthropods (probably crustaceans) as intermediate hosts.

Bilqees et al. [4] established a new genus and species *Pseudomazzia macrolabiata* Bilqees, Ghazi & Haseeb, 2005 based on nematode specimens found in the intestine of the marine fish *Pomadasys olivaceus* (Day) (Haemulidae, Perciformes) from off the Karachi coast in Pakistan. The authors considered this nematode to be related to *Mazzia* Khalil & Vogelsang, 1932, a genus represented by two species parasitizing dasypodid mammals in Argentina and belonging to the spiruroid family Spirocercidae Chitwood & Wehr, 1932 [3]. However, the illustrations of the cephalic end of *P*. *macrolabiata* are reminiscent of a species of *Rasheedia* and this possibility is also supported by the type of the host (marine fish). Unfortunately, both the description and illustrations of these nematodes are poor and confusing, making it necessary to consider *Pseudomazzia* Bilqees, Ghazi & Haseeb, 2005 and *P*. *macrolabiata* to be *nomina dubia*.

### Key to species of *Rasheedia*

1. Body length of male and gravid female more than 10 mm. Glandular oesophagus more than 7 mm long. Parasitic in Polynemidae in the Indo-West Pacific ................................ 2

– Body length of male and gravid female less than 10 mm. Glandular oesophagus less than 4 mm long. Parasitic in Mullidae and Sparidae in the eastern North Atlantic or the western South Pacific ..................................................................................................................... 3

2. Left spicule 260 μm long, length of right spicule 90 μm; length ratio of spicules 1:2.9. Tail tip of female with three small points. Parasites of an unknown fish and *Eutheronema tetradactylum* in the Arabian Sea (Pakistan) ...................................................... *R*. *inglisi*

– Left spicule 320–410 μm long, length of right spicule 105–120 μm; length ratio of spicules 1:3.39. Tail tip of female with single point. Parasitic in *Eutheronema tetradactylum* in the South China Sea (South Vietnam) ................................................................ *R*. *deblocki*

3. Four anteriorly protrusible oesophageal lobes present. Middle part of muscular oesophagus not expanded. Left spicule 381 μm long, length of right spicule 87 μm. Female not known. Parasitic in *Parupeneus indicus* in the western South Pacific (New Caledonia) ............................................................................................. *R*. *novaecaledoniensis* n. sp.

– Two or three anterior protrusible oesophageal lobes present. Middle part of muscular oesophagus expanded or not expanded. Spicules longer ............................................... 4

4. Three anteriorly protrusible oesophageal lobes present. Middle part of muscular oesophagus not expanded. Left spicule 395 μm long, length of right spicule 100 μm; length ratio of spicules 1:3.95. Egg shell without additional thick layer. Parasitic in *Pseudupeneus prayensis* in the eastern North Atlantic (Senegal) ........ *R*. *pseudupenei*

– Two anteriorly protrusible oesophageal lobes present. Middle part of muscular oesophagus distinctly expanded. Left spicule 435–489 μm long, length of right spicule 153–156 μm; length ratio of spicules 1:2.79–3.20. Shell of most developed eggs with additional thick layer. Parasitic in *Parupeneus heptacanthus* and *Dentex fourmanoiri* in the western South Pacific (New Caledonia) ................................................................ *R*. *heptacanthi* n. sp.

## Conflict of interest

The Editor-in-Chief of Parasite is one of the authors of this manuscript. COPE (Committee on Publication Ethics, http://publicationethics.org), to which Parasite adheres, advises special treatment in these cases. In this case, the peer-review process was handled by an Invited Editor, Jérôme Depaquit.
